# Investigation of the trend of drowning deaths in the provinces of Iran, 2017-2020 

**Published:** 2022-12

**Authors:** Batoul Rabbani, Amin Ataey, Hossein Kazemeini, Mahinsadat Azimi, Mostafa Rezaei, Elham Rashidian, Ardeshir Khosravi

**Affiliations:** ^*a*^ Center for Health Network, Vice Chancellery for Health, Ministry of Health and Medical Education, Tehran, Iran.

## Abstract

**Background:**

Drowning is the third leading cause of death due to unintentional injuries worldwide and accounts for 7% of all injury‑related deaths. The aim of this study was to investigate the trend of deaths due to drowning in the provinces of Iran from 2017 to 2020.

**Methods:**

This is a descriptive study in which the data on deaths due to drowning during 2017-2020 were extracted from the Death Registration System of the Ministry of Health and Medical Education (using the W65-W74 code based on the International Classification of Diseases ICD-10). We used population data from the population and housing census (2016) and population estimates for the years after the census, which were prepared and published by the Statistics Center of Iran.

**Results:**

According to the reports, 862, 853, 1126, and 1028 deaths due to drowning were registered in the Death Registration System of the Ministry of Health and Medical Education in 2017, 2018, 2019, and 2020, respectively. In all these years, 82% of deaths occurred in men. During these four years, 92-93% of these deaths occurred among Iranians, and 53-57% of them were in urban areas. The proportion of deaths according to age groups was 10% in less than 5-year-old children, 22-27% in the 5- to 17-year-old, and 24-29% in the 18- to 29-year-old ones. Death due to drowning ranked third in the age group of 5-14 years in 2019 and 2020 and fourth in the age group of 1-4 years in 2018 and 2020. In 2017, the highest rate of deaths (per 100000 population) due to drowning belonged to Khuzestan province (2.63). In 2018 and 2019, Bushehr province (2.91-3.19), and in 2020 Hormozgan province (3.20) had the highest number of deaths due to drowning.

**Conclusions:**

Depending on the different circumstances, at-risk population groups, and geographical conditions, provincial authorities should plan on preventing deaths and complications due to drowning.

**Keywords:**

Drowning, Burden of drowning, Iran, Trend

